# Supplementary material for: Microbe-Derived Antioxidants Alleviate Liver and Adipose Tissue Lipid Disorders and Metabolic Inflammation Induced by High Fat Diet in Mice
Source: Int J Mol Sci. 2023 Feb 7;24(4):3269. doi: 10.3390/ijms24043269 (PMC9965291; doi:10.3390/ijms24043269)
Supplement: Supplementary file 1 [file ijms-24-03269-s001.zip › ijms-2078781-supplementary.pdf]

Table S1. Primers used in current study

Gene	Accession Number	Primer sequence(5'—3')
β-actin	NM_0073 93.5	F:CTAAGGCCAACCGTGAAAAG<br>R:TACATGGCTGGGGTGTTGA
TERT	NM_001172073.1	F:CGCTACAACGCTTGGTC<br>R:AGATGAGGCTGGGAACG
NLRP3	XM_0361 56549.1	F:AGAAGAGACCACGGCAGAAG<br>R:CCTTGGACCAGGTTCAGTGT
ASC	NM_0232 58.4	F:GACAGTGCAACTGCGAGAAG<br>R:CGACTCCAGATAGTAGCTGACAA
Caspase1	NM_0012 78601.1	F:CTTGGAAATAGCTCCCAGAA<br>R:CATTTGGGAACTTCTCATCC
IL-18	XM_0361 54618.1	F:CCTTTGAGGCATCCAGGACA<br>R:CACACCACAGGGGAGAAGTG
IL-1β	NM_0083 61.4	F:TGCCACCTTTTGACAGTGATG<br>R:AAGGTCCACGGGAAAGACAC
FAS	XM_0302 45556.1	F:TCCTGGAACGAGAACACGATCT<br>R:GAGACGTGTCACTCCTGGACTTG
CPT1b	NM_0099 48.2	F:ACGGGTGGATGTTCGAGATG<br>R:GGCAGTGACGTTTGGAAGCT
PPARα	XM_0302 48424.2	F:ACGCATGTGAAGGCTGTAAGG<br>R:CGACAGACAGGCACTTGTGAAA
PPARγ	XM_0173 21455.3	F:TTTCAAGGGTGCCAGTTTGG<br>R:CCATCTTTATTCATCAGGGAGGC
ACCα	NM_1333 60.3	F:TGTCCGCACTGACTGTAACCAC<br>R:TAGCCAGACTCGTTTGTCAGGA
CD36	XM_0302 54088.1	F:TGAGACTGGGACCATTGGTGAT<br>R:CCCAAGTAAGGCCATCTCTACCAT
FABP1	NM_0173 99.5	F:CAATAGGTCTGCCCGAGGAC<br>R:CACCTTCCAGCTTGACGACT
HSL	XM_0302 42180.1	F:AAGGACTCACCGCTGACTTCC<br>R:GCCTGTCTCGTTGCGTTTGTA
ATGL	NM_0258 02.3	F:TCCGTGGCTGTCTACTAAAGA<br>R:TGGGATATGATGACGTTCTCTCC

Tables S2. The ingredient of diet

**product**	**D12492**		**D12492J**	
**Ingredient**	**gm**	**kcal**	**gm**	**kcal**
Casein, 80 Mesh	200	800	200	800
L-Cystine	3	12	3	12
Corn Starch	0	0	506.2	2024.8
Maltodextrin 10	125	500	125	500
Sucrose	68.8	275	68.8	275.2
Cellulose, BW200	50	0	50	0
Soybean Oil	25	225	25	225
Lard	245	2205	20	180
Mineral Mix S10026	10	0	10	0
Dicalcium Phosphate	13	0	13	0
Calcium Carbonate	5.5	0	5.5	0
Potassium Citrate, 1 H2O	16.5	0	16.5	0
Vitamin Mix V10001	10	40	10	40
Choline Bitartrate	2	0	2	0
FD&C Blue Dye #1	0.05	0	0.01	0
FD&C Yellow Dye #5	0	0	0.04	0

CON and CMA were fed conventional diet (10% fat, 70% Carbohydrate, 20% Protein, 3.85kcal/g, D12492J), while HFD and HMA were fed high-fat diet (60% fat, 20% Carbohydrate, 20% Protein, 5.24kcal/g, D12492).Conversion according to the International System of Units (1kcal=4.185×1000 J): conventional diet energy: 1.61×10^{7} J/kg, high-fat diet energy: 2.19×10^{7} J/kg.
